# ATF5 promotes malignant T cell survival through the PI3K/AKT/mTOR pathway in cutaneous T cell lymphoma

**DOI:** 10.3389/fimmu.2023.1282996

**Published:** 2023-12-22

**Authors:** Mengzhou Cao, Pan Lai, Xiangjun Liu, Fengjie Liu, Yao Qin, Ping Tu, Yang Wang

**Affiliations:** ^1^ Department of Dermatology and Venereology, Peking University First Hospital, Beijing, China; ^2^ Beijing Key Laboratory of Molecular Diagnosis on Dermatoses, Beijing, China; ^3^ National Clinical Research Center for Skin and Immune Diseases, Beijing, China

**Keywords:** cancer pathogenesis, transcription factor, malignant T cell, phosphoinositide 3-kinase, endoplasmic reticulum stress

## Abstract

**Backgrounds:**

Cutaneous T cell lymphoma (CTCL) is a non-Hodgkin lymphoma characterized by skin infiltration of malignant T cells. The biological overlap between malignant T cells and their normal counterparts has brought obstacles in identifying tumor-specific features and mechanisms, limiting current knowledge of CTCL pathogenesis. Transcriptional dysregulation leading to abnormal gene expression profiles contributes to the initiation, progression and drug resistance of cancer. Therefore, we aimed to identify tumor-specific transcription factor underlying CTCL pathology.

**Methods:**

We analyzed and validated the differentially expressed genes (DEGs) in malignant T cells based on single-cell sequencing data. Clinical relevance was evaluated based on progression-free survival and time to next treatment. To determine the functional importance, lentivirus-mediated gene knockdown was conducted in two CTCL cell lines Myla and H9. Cell survival was assessed by examining cell viability, colony-forming ability, *in-vivo* tumor growth in xenograft models, apoptosis rate and cell-cycle distribution. RNA sequencing was employed to investigate the underlying mechanisms.

**Results:**

Activating transcription factor 5 (ATF5) was overexpressed in malignant T cells and positively correlated with poor treatment responses in CTCL patients. Mechanistically, ATF5 promoted the survival of malignant T cells partially through the PI3K/AKT/mTOR pathway, and imparted resistance to endoplasmic reticulum (ER) stress-induced apoptosis.

**Conclusions:**

These findings revealed the tumor-specific overexpression of the transcription factor ATF5 with its underlying mechanisms in promoting tumor survival in CTCL, providing new insight into the understanding of CTCL’s pathology.

## 1 Introduction

Cutaneous T cell lymphomas (CTCLs) are a heterogeneous group of non-Hodgkin lymphomas, including mycosis fungoides (MF), Sézary syndrome (SS), and primary cutaneous CD30-positive lymphoproliferative disorders (CD30+LPDs) (1). Despite evident heterogeneity and interconnections among its subtypes, the mechanisms driving the development and progression of CTCL have not been fully elucidated. CTCL is characterized by cutaneous infiltration of monoclonally proliferative skin-homing T cells, alongside polyclonal reactive T cells triggered by inflammation and malignancy (2). The biological overlap between malignant T cells and normal T cells within cutaneous lesions presents challenges in unraveling the tumor-specific pathogenesis.

Efforts in recent decades have aimed to discover molecular markers that are specifically expressed in malignant T cells, as opposed to benign T cells. Loss of pan-T antigens, such as CD7 (3) and CD26 (4) was observed in circulating CD4+ T cells with morphological aberrance and has been applied in clinical diagnosis (5). The functional importance of these biomarkers to CTCL development, however, has rarely been depicted in previous studies. Immunostaining of thymocyte selection-associated high-mobility group box (TOX) was proposed to distinguish between CTCL, especially early-stage MF, and benign inflammatory dermatosis (BID) (6). However, the reactive T cells in BID commonly express TOX, limiting the development of targeted therapy (7). Other malignant T cell-specific molecular markers, such as KIR3DL2 (CD158k), have shown promising efficacy as therapeutic targets, tending to be expressed in Sézary cells or in patients with late-stage MF (8, 9).

Transcription factors bind to specific DNA sequences in the enhancers and promoters to regulate transcription, a process converting genetic information from DNA to mRNA (10). The well-organized expression of genes, under the proper regulation of transcription factors, is crucial for maintaining normal cellular structure and function (11). On the other hand, dysregulated transcription factors result in abnormal gene expression and disrupted cellular homeostasis, representing a key characteristic in the development of cancer (12). The aberrant gene expression pattern led by transcriptional dysregulation contributes to tumor initiation, progression and drug resistance (13–18). Therefore, understanding dysregulated transcription factors in cancer provides valuable insights into the mechanisms driving tumorigenesis and helps develop efficient treatment strategies (19, 20). In recent years, single-cell-based transcriptome sequencing combined with TCR sequencing has enabled the identification of malignant T cells at the single-cell level, offering a new perspective into the heterogeneous dysregulation of transcription within CTCL skin lesions. Based on previously reported single-cell RNA sequencing (scRNA-seq) data from our group (21), we identified ATF5 as a prosurvival transcription factor specifically expressed in malignant T cells in CTCL and unraveled its underlying mechanism.

## 2 Materials and methods

### 2.1 Identification for differentially upregulated genes in malignant T cells

Single-cell RNA sequencing, cell type identification and gene expression comparison were conducted as described in our previous research (21). Specifically, data from 10× Genomics RNA and TCR sequencing of 13 samples were reanalyzed. Large-scale CNVs were inferred using the infercnv package. Malignant T cells were differentiated from normal T cells based on CNVs and TCR clonotype. Differential RNA expression was determined between malignant T cells and their respective normal CD4+ T cells or CD8+ T cells from each sample. For the 11 samples of CD4+ CTCL, normal CD4+ T cells were referred to as the normal T and compared with their malignant T cells. Similarly, for the 2 samples of CD8+ CTCL, normal CD8+ T cells were referred to as normal T and compared with malignant T cells. Differentially upregulated genes were defined by a fold change>1.5 and adjusted *p* value<0.05.

### 2.2 CTCL cell lines and primary cells

Human CTCL cell lines H9, Myla, HH, Hut78, Mac1, Mac2A, PB2B, and Sz4 and the acute T-cell leukemia cell line Jurkat were cultured in RPMI-1640 medium with 10% fetal bovine serum, 100 U/ml penicillin, and 0.1 mg/ml streptomycin. Human CTCL cell line MJ was cultured in IMDM with 20% fetal bovine serum, 100 U/ml penicillin, and 0.1 mg/ml streptomycin.

Peripheral blood mononuclear cells (PBMCs) were obtained from 2 healthy donors by Ficoll-Paque PLUS (GE Healthcare). Peripheral blood CD4+ T cells were purified from PBMCs with a CD4+ T cell isolation kit (Biolegend).

### 2.3 Cell treatment

Cells were seeded in 12-well plates at a density of 1×10^5/ml. Hypoxia was induced by adding liquid paraffin on top to seal the culture medium. Drug concentrations were labeled in graphs or as follows: hydrogen peroxide (H_2_O_2_), 200 μM; low glucose, 1 mM; low serum, 1% fetal bovine serum; paraquat, 100 μM; thapsigargin, 5 nM for H9, 200 nM for Myla; tunicamycin, 0.5 μM for H9, 1 μM for Myla.

### 2.4 Lentivirus-mediated gene knockdown and overexpression

Two lentivirus short hairpin RNA (shRNA) vectors were constructed using the GV644 vector (GeneChem) and two independently designed oligonucleotides encoding shRNAs against ATF5. Control vectors were constructed with oligonucleotides encoding scrambled shRNA. The ATF5 lentivirus overexpression vector was constructed by ligating full-length human ATF5 (NM_001193646) cDNA into the GV643 vector (GeneChem). Cells were transfected according to the manufacturer’s instructions. shRNA oligonucleotide sequences are listed in **Supplementary Table S1**.

### 2.5 Cell viability and apoptosis assays

For cell viability in normal culture, cells were cultured for 96h, and MTS-based cell viability was measured at 0h, 24h, 48h, 72h, and 96h using Cell Viability Colorimetric Assay Kit (Promega). Relative cell viability at each time point was normalized by reads at 0h. For LY294002 treatment, cells of shATF5 and shNC groups were treated and cultured for 48h. For each group, cell viability value after drug treatment (OD) was normalized by the cell viability value of the counterpart untreated cells (OD0μM), calculated as OD/OD0μM. This calculation obtains the relative cell viability, as shown in the bar graph.

For apoptosis, cells were stained with Annexin V-APC (BD Pharmingen) and apoptosis rates were quantified by flow cytometry. For drug-induced specific apoptosis, cells of shATF5 and shNC groups were treated as indicated. For each group, apoptosis rates with and without drug treatment were measured. Specific apoptosis was calculated using the formula: (percentage of apoptosis in treated cells - percentage of spontaneous apoptosis in untreated cells)/(100 - percentage of spontaneous apoptosis in untreated cells) (22, 23).

### 2.6 Lactate production assays

Lactate production assays were conducted using a lactate assay kit (Yuanye) and measured by absorbance (OD) at 540 nm. Cells of shATF5 and shNC groups were seeded at a density of 2×10^5^/ml. The lactate concentration (mmol/L) in the cell culture supernatant was calculated as [OD-OD(culture medium without cells)]/[OD-OD(standard)] × standard concentration × dilution ratio. Lactate production at 48 h was calculated as (lactate concentration of cell supernatant) – (lactate concentration of culture medium without cells).

### 2.7 Bioinformatic analysis

Functional annotation was conducted on DAVID (https://david.ncifcrf.gov/). Enriched pathways are listed in **Supplementary Table S2**. The promoter sequence from -2 kb to +100 bp of the PIK3AP1 transcription start site was obtained from NCBI and was subjected to Jaspar (https://jaspar.genereg.net) for motif searching. The retrieved results are listed in **Supplementary Table S3**.

### 2.8 Statistics

Statistics were analyzed with GraphPad Prism (version 8.4.3) and R (version 4.0.3). Significant differences between two independent groups were analyzed by Student’s two-tailed t test. The correlation between the expression levels of two genes was determined by Spearman analysis. Kaplan−Meier analysis was performed and plotted using the survminer package (https://CRAN.R-project.org/package=survminer). A *p* value<0.05 was considered statistically significant.

## 3 Results

### 3.1 ATF5 is overexpressed in malignant T cells and is associated with unfavorable treatment responses in CTCL

To identify dysregulated transcription factors in malignant T cells in cutaneous T-cell lymphoma (CTCL), we conducted an analysis on the differentially expressed genes (DEGs) between malignant T cells and benign reactive T cells based on a single-cell RNA sequencing (scRNA-seq) dataset from our previous study (21). Within this dataset, the differentially upregulated genes (fold change>1.48, adjusted p value<0.05) were sorted based on the frequency of occurrence across the samples. The transcription regulator TOX, which has previously been reported as a marker for differential diagnosis between CTCL and benign inflammatory dermatoses (6), was found to be specifically upregulated in the malignant T cells in 4 out of 13 samples. Therefore, genes upregulated in 5 samples or more were defined as frequently recurrent DEGs in malignant T cells and are listed in **Table 1**. Among these genes, transcription factors *ATF5* and *BHLHE40* were identified. Other frequently recurrent DEGs were involved in various cellular processes, including cellular interactions (*LGALS1*, *LGALS3*, *SPINT2*, *CXCL13*, *LAIR2*, *ITM2A*, *CLU*), cell metabolism (*GAPDH*, *TPI1*, *SMS*), signal transduction (*S100A6*, *S100A11*, *S100A10*, *S100A4*, *ANXA2*), cytoskeleton (*LMNA*, *TUBA1B*, *TAGLN2*), mitosis (*STMN1*, *PTTG1*), cellular transportation (*MFSD10*), cancer-testis antigens (*PAGE5*, *CST7*), redox reaction (*PRDX1*), protein folding (*PPIA*) and lncRNA (*AC006129.4*).

**Table 1:** Frequently recurrent DEGs from scRNA-seq.

Functional classification	Gene symble	Number of samples*	Average fold change#
Transcription factor	ATF5	6	1.81
	BHLHE40	5	1.77
Cellular interaction	LGALS1	9	2.93
	LGALS3	6	2.20
	SPINT2	6	1.99
	CXCL13	5	4.42
	LAIR2	5	1.77
	ITM2A	5	1.63
	CLU	5	1.48
Cell metabolism	GAPDH	7	2.33
	TPI1	7	2.09
	SMS	5	1.79
signal transduction	S100A6	8	1.78
	S100A11	8	1.48
	S100A10	6	1.56
	S100A4	5	1.68
	ANXA2	5	1.64
cytoskeleton	LMNA	6	2.04
	TUBA1B	5	1.96
	TAGLN2	5	1.51
mitosis	STMN1	5	1.90
	PTTG1	5	1.84
cellular transportation	MFSD10	5	1.59
cancer-testis antigen	PAGE5	5	1.89
	CST7	5	1.60
redox reaction	PRDX1	5	1.92
Protein folding	PPIA	6	1.49
IncRNA	AC006129.4	5	1.76

*the number of samples where this gene differentially upregulated in malignant T cells (fold change>1.48 and p.adj<0.05). #Average fold change was determined between all the malignant T cells and normal T cells from 13 samples.

**Figure 1:**
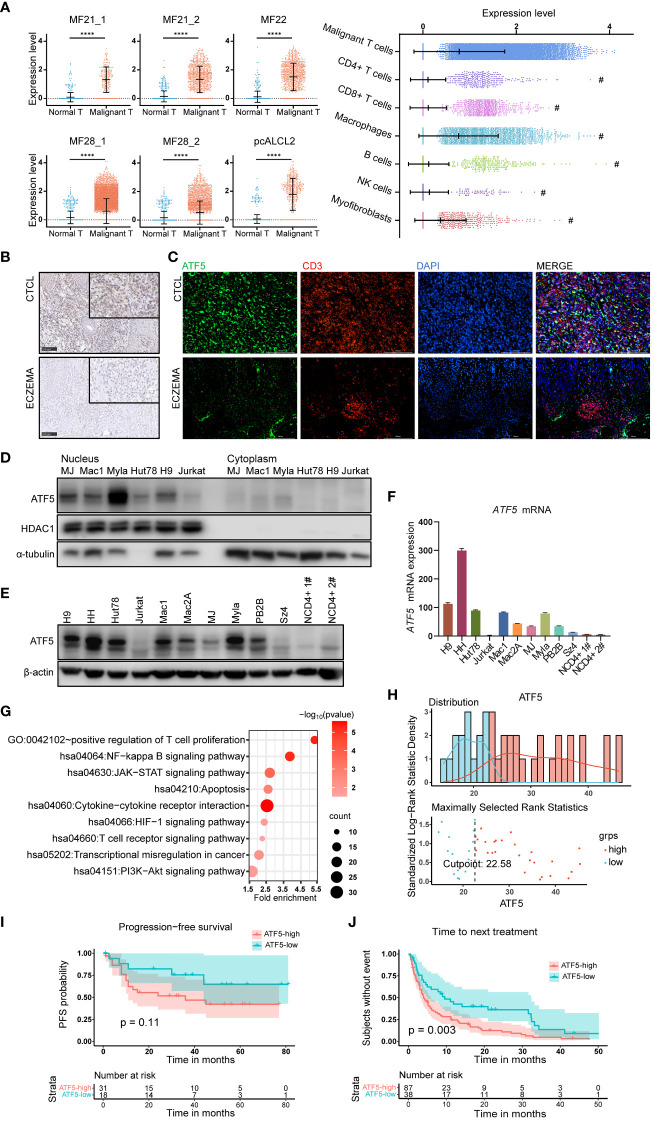
ATF5 is highly expressed in cutaneous T cell lymphoma and associates with poor treatment response. **(A)** Normalized ATF5 expression in various cell types in 6 samples from 4 CTCL patients who have higher ATF5 expression in malignant T cells. Malignant T: malignant T cells, Normal T: normal T cells **(B)** Representative images of immunohistochemical staining of ATF5 in paraffin-embedded tissues from CTCL patients and eczema patients. Original magnification ×200, insets ×400, scale bar=50 μm. **(C)** Representative images of immunofluorescence costaining of ATF5 (green) and CD3 (red) in paraffin-embedded tissues from CTCL patients and eczema patients. DAPI (blue) was used to visualize cell nuclei. Original magnification ×200, scale bar=200 μm. **(D)** ATF5 protein expression in nuclear and cytoplasmic lysates of CTCL cell lines and Jurkat cells. **(E, F)** Expression levels of ATF5 protein **(E)** and mRNA **(F)** in 9 CTCL cell lines, Jurkat cells, and CD4+ T cells from 2 normal controls (NCD4+ 1#, NCD4+ 2#). ATF5 mRNA expression levels were normalized to GAPDH. **(G)** Annotated pathways from genes (n=873, *p*<0.05) positively correlated with ATF5 expression in our previously reported CTCL cohort with RNA sequencing (FDR<0.25, *p*<0.05). **(H)** ATF5 expression distribution and cut point for prognosis and treatment analysis in the CTCL cohort. A total of 49 patients with regular follow-up were recruited from March 2010 to December 2018. The median (range) follow-up duration was 32 (1–81) months. **(I)** Kaplan−Meier survival analysis of PFS (n=49 patients). Progression-free survival information was collected for each patient from the time of biopsy to the time of disease progression. **(J)** Kaplan−Meier survival analysis of TTNT (n=125 treatment episodes). Data are shown as mean ± SD and representative of at least three independent experiments. ****P<0.0001.

Notably, *ATF5*, a member of the basic leucine zipper (bZip) family (24), was the most consistently upregulated transcription factor across multiple samples. ATF5 was upregulated in malignant T cells from 6 lesions in 4 patients, while its expression remained low in normal T cells (**Figure 1A**). We also compared the expression levels of ATF5 across different cell types in the tumor microenvironment (25–27). We found that malignant T cells exhibit the highest expression of ATF5 compared to other cell types, while macrophages also demonstrate significant levels of ATF5 expression, the clinical significance of which is yet to be determined (**Figure 1A**). In the cases where more than one biopsy was taken (MF21 and MF28) (21), the expression levels of ATF5 in malignant T cells from different skin lesions were similar, regardless of biopsy site or lesion duration (**Figure 1A**). This observation, consistent with a previous study (28), indicated that the overexpression of ATF5 was more likely due to intrinsic malignant traits rather than a lesion-specific environment. Immunohistochemical staining of ATF5 (**Figure 1B**) and immunofluorescent co-staining with CD3 (**Figure 1C**) on lesional biopsies confirmed the increased expression of ATF5 protein in atypical CD3+ T cells from CTCL patients compared to normal CD3+ T cells from benign inflammatory dermatoses patients. Consistent with its main role as a transcription factor, ATF5 protein was predominantly detected in the nuclear component of patient samples (**Figures 1B, C**; **Supplementary Figure 1**) and CTCL cell lines (**Figure 1D**). Furthermore, CTCL cell lines showed heterogeneous, yet consistently higher, expression of ATF5 at both the mRNA and protein levels, compared to peripheral blood CD4+ T cells from healthy donors and the non-CTCL T-cell leukemic cell line Jurkat (**Figures 1E, F**).

Considering the relatively specific expression of ATF5 in malignant T cells, we evaluated the clinical relevance of ATF5 expression in a cohort of 49 tumor-stage CTCL patients with bulk RNA sequencing data which was published by our group previously (29). Genes positively correlated with ATF5 expression (n=873 genes with r>0, *p*<0.05) were enriched in pathways (FDR<0.25, *p* value<0.05) associated with CTCL development, including positive regulation of T cell proliferation, NF-kappa B signaling pathway, T cell receptor signaling pathway, JAK/STAT signaling pathway and PI3K/AKT pathway (**Figure 1G**; **Table S2**). These findings indicated that the upregulation of ATF5 in malignant T cells was implicated in the pathogenesis of CTCL. Patient prognosis was assessed through progression-free survival (PFS), while treatment response was evaluated by time to next treatment (TTNT). In this cohort, the median (range) follow-up duration was 32 (1–81) months. A total of 125 treatment episodes were administered during follow-up and were included in our analysis. Patients were divided into ATF5-high or ATF5-low group based on the transcriptional ATF5 levels. The cutoff value was determined by the surveminer package according to ATF5 expressions and the PFS data (**Figure 1H**). Patients in the ATF5-high group exhibited a tendency towards shorter progression-free survival, although the difference was not statistically significant (**Figure 1I**), which could be attributed to the fact that all the patients from this cohort were in the tumor stage. Notably, patients in the ATF5-high group experienced shorter durations in different lines of therapy (**Figure 1J**). Given the requirement for long-term medical treatment in CTCL patients, this rapid transition to subsequent therapeutic regimen indicated that patients with high ATF5 expression benefited less from current treatments, mainly due to unsatisfactory therapeutic effects (30).

Collectively, these results indicated the specific overexpression of the transcription factor ATF5 in malignant T cells among CTCL patients, which were associated with inferior therapeutic outcomes.

### 3.2 ATF5 knockdown hinders tumor survival by promoting cell apoptosis in CTCL lines

**Figure 2:**
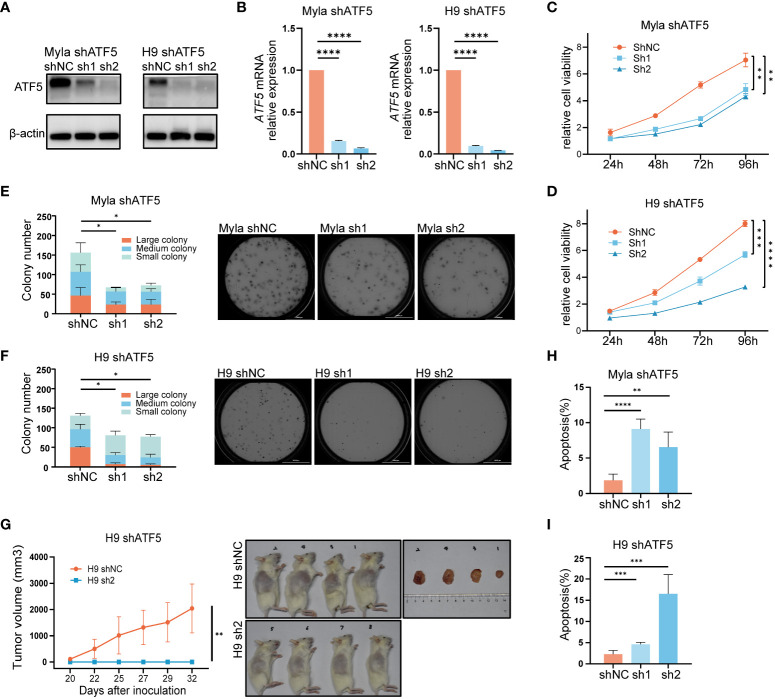
Interference with ATF5 expression inhibits CTCL cell growth. **(A, B)** Suppression of ATF5 protein **(A)** and mRNA **(B)** expression in Myla and H9 cell lines by lentivirus (shATF5) with two independent shRNA sequences (sh1, sh2). Cells transfected with scramble RNA were used as a control (shNC). Relative ATF5 mRNA expression was normalized to GAPDH and shNC. **(C, D)** Relative cell viability of control (shNC) and ATF5-KD (sh1, sh2) Myla and H9 cells normalized to 0 h. Student’s t-test was used to compare the relative cell viability of shNC vs. sh1 and shNC vs. sh2 at 96h. **(E, F)** Number of colonies of different sizes formed by control (shNC) and ATF5-KD (sh1, sh2) Myla **(E)** and H9 **(F)** cells in CFC assays. Representative pictures taken under microscope are shown on the right. Large colony: diameter > 60μm; medium colony: diameter between 30-60 μm; small colony: diameter < 30μm **(G)** Tumor sizes of xenograft models injected with control (shNC) and ATF5-KD (sh1, sh2) H9 cells. Tumor sizes were measured at the times indicated. Images of xenograft mice and tumors taken at the end of the experiment are shown on the right. **(H, I)** Apoptosis rate of control (shNC) and ATF5-KD (sh1, sh2) Myla **(H)** and H9 **(I)** cells. Data are shown as mean ± SD and representative of at least three independent experiments. *P<0.05, **P<0.01, ***P<0.001, ****P<0.0001.

Given its specific overexpression in malignant T cells, we sought to investigate whether ATF5 contributed to malignant behaviors in CTCL. To understand the role of ATF5 in CTCL, we employed lentivirus-mediated shRNA transduction to suppress ATF5 expression in CTCL cell lines Myla and H9, with scrambled shRNA as a control. The suppression of ATF5 expression was confirmed at both the protein and mRNA levels (**Figures 2A, B**). MTS-based viability assays demonstrated reduced expansion rate in ATF5-knockdown (ATF5-KD) Myla and H9 cells (**Figures 2C, D**). In the colony-forming cell (CFC) assays, ATF5-KD Myla and H9 cells showed decreased colony number and size in semisolid cultures, indicating an impaired colony-forming ability (**Figures 2E, F**). Furthermore, ATF5 knockdown significantly impeded tumor growth in a xenograft model using H9 cells. ATF5-KD H9 cells failed to form subcutaneous tumors in the grafted sites, while control cells formed considerable tumors (**Figure 2G**). Therefore, ATF5 knockdown inhibited the survival of malignant T cells, prompting a further investigation of apoptosis and proliferation characteristics. Apoptosis rates were significantly increased in ATF5-KD Myla and H9 cells (**Figures 2H, I**), while cell cycle distributions were not remarkably affected by ATF5 suppression (**Supplementary Figure 2**). Thus, the inhibition of tumor survival following ATF5 knockdown was primarily due to increased apoptosis rate.

These results confirmed that ATF5 promoted tumor survival and contributed to the tumor progression of CTCL.

### 3.3 ATF5 protects malignant T cells against endoplasmic reticulum stress-induced apoptosis

**Figure 3:**
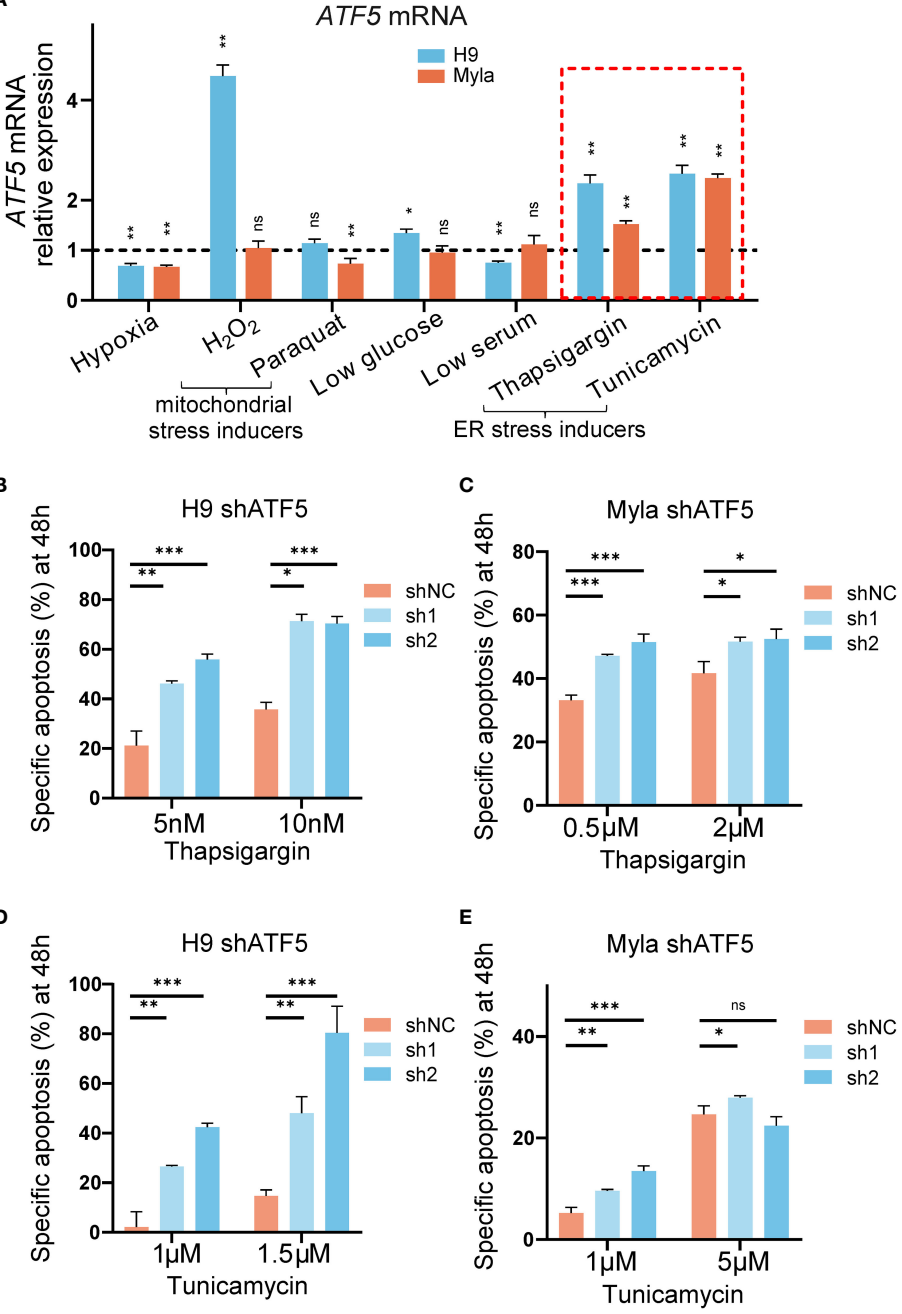
ATF5 is upregulated under ER stress and protects cells from ER stress-induced apoptosis. **(A)** Relative ATF5 mRNA expression in Myla and H9 cells treated for 24h. Relative expression was normalized to GAPDH and vehicle. Relative ATF5 expressions of vehicle groups were normalized as 1 and indicated by the black dashed line. The red dashed box highlights ER stress inducers treatment. **(B, C)** Specific apoptosis of control (shNC) and ATF5-KD (sh1, sh2) H9 **(B)** and Myla **(C)** cells treated with thapsigargin for 48 h. **(D, E)** Specific apoptosis of control (shNC) and ATF5-KD (sh1, sh2) H9 **(D)** and Myla **(E)** cells treated with tunicamycin for 48 h. Data are presented as mean ± SD and representative of at least three independent experiments. ns, no significance, *P<0.05, **P<0.01, ***P<0.001.

We noticed that the suppression of tumor survival induced by ATF5 knockdown was more prominent in semisolid cultures and xenograft models than in liquid cultures. Liquid-cultured cells are provided with moderate culture density, sufficient nutrients, and a suitable environment, whereas tumor cells face monoclonal expansion pressures in semisolid cultures and experience nutrient deficiency and hypoxia in xenograft models. Accumulating evidence suggests that ATF5 acts as a prosurvival factor under cellular stress (31–33). To assess the involvement of ATF5 in the stress response in CTCL, we exposed the liquid-cultured CTCL cell lines Myla and H9 to various stress-inducing conditions and assessed their impact on ATF5 expression. Both Myla and H9 cells were treated with various stressors, including hypoxia, mitochondrial stress inducers (H_2_O_2_, paraquat), low glucose, low serum and ER stress inducers (thapsigargin, tunicamycin). ATF5 expression showed a slight decline under hypoxia, while changes in expression were inconsistent between Myla and H9 cells under mitochondrial stress, low glucose, and low serum (**Figure 3A**). Notably, ATF5 consistently upregulated when treated with the ER stressors thapsigargin and tunicamycin in both cell lines (highlighted by the red dashed box in **Figure 3A**). We further treated control and ATF5-KD cells with or without ER stress inducers and calculated ER stress-induced specific apoptosis (22). Notably, ATF5-KD Myla and H9 cells showed a marked increase in specific apoptosis induced by thapsigargin and tunicamycin (**Figures 3B–E**), indicating that ATF5 protects malignant T cells against ER stress-induced apoptosis in CTCL.

Together, these results demonstrated that ATF5 protected against ER stress-induced apoptosis in CTCL.

### 3.4 ATF5 promotes CTCL partially through the PI3K/AKT/mTOR pathway

**Figure 4:**
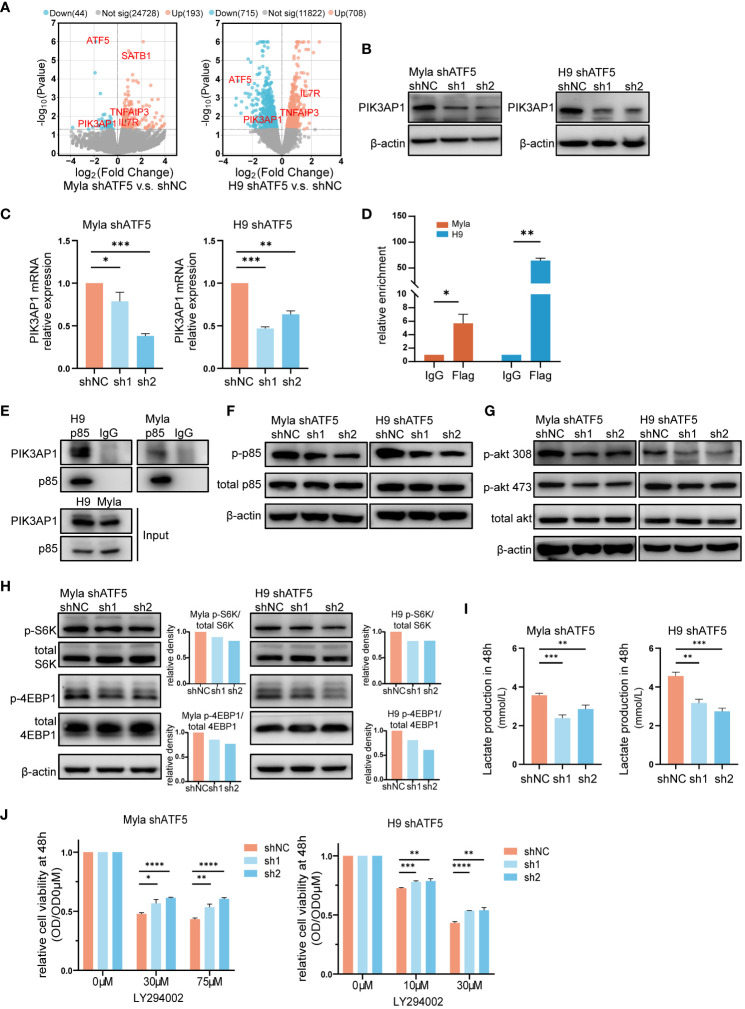
The PI3K/AKT/mTOR pathway partially accounts for ATF5-promoted CTCL survival. **(A)** Volcano plot of upregulated (red) and downregulated (blue) DEGs (|fold change|>0 and *P*<0.05) between control (shNC) and ATF5-KD (shATF5) H9 and Myla cells. RNA was prepared 72 hours after transduction, following the validation of ATF5 knockdown. **(B, C)** Downregulation of PIK3AP1 protein **(B)** and mRNA **(C)** levels in control (shNC) and ATF5-KD (sh1, sh2) Myla and H9 cells. Relative PIK3AP1 mRNA expression was normalized to GAPDH and shNC. **(D)** Enrichment of ATF5 protein at the PIK3AP1 promoter in Myla and H9 cell lines stably expressing Flag-ATF5. **(E)** Immunoprecipitation in Myla and H9 cells using a p85 antibody or normal rabbit IgG with protein A/G agarose. PIK3AP1 and p85 were identified. **(F-H)** Phosphorylated p85, Akt, S6K and 4EBP1 in control (shNC) and ATF5-KD (sh1, sh2) Myla and H9 cells. The relative density of phosphorylated protein was normalized to total protein and control (shNC). Protein was prepared 72 hours after transduction, following the validation of ATF5 knockdown. **(I)** Lactate production at 48 h in control (shNC) and ATF5-KD (sh1, sh2) Myla and H9 cells. **(J)** Relative cell viability of control (shNC) and ATF5-KD (sh1, sh2) Myla and H9 cells after LY294002 treatment. Cell viability of the treated cells was normalized to that of the untreated cells at 48 h. Data are presented as mean ± SD and representative of at least three independent experiments. *P<0.05, **P<0.01, ***P<0.001, ****P<0.0001.

To investigate the mechanisms underlying the effects of ATF5 on CTCL cells, we performed transcriptome sequencing and identified differentially expressed genes (DEGs) between control and ATF5-KD Myla and H9 cells (**Figure 4A**). Since ATF5 primarily functions as a transcription factor, we focused on genes that were downregulated in both Myla and H9 cells upon ATF5 knockdown. Among these genes, *PIK3AP1* exhibited the most significant reduction in expression (**Figures 4A–C**). PIK3AP1 is an adaptor protein required for the activation of PI3K/AKT signaling (34). PIK3AP1 promotes PI3K activity by directly binding to p85, enabling the phosphorylation of p85 by the intracellular segment of tyrosine receptor, and subsequently activates downstream PI3K/AKT pathway (34–36). PI3K phosphorylates AKT at Thr308 and Ser473, subsequently activating mTORC1 (37). The PI3K/AKT/mTOR activity, under the regulation of PIK3AP1, regulates T-cell homeostasis (35, 36), as well as tumor survival and metabolic adaptation (37, 38). Genes positively correlated with ATF5 in CTCL patients were enriched in the PI3K/AKT pathway (**Figure 1G**), which was essential for CTCL survival (39).

Therefore, we investigated whether the overexpressed ATF5 activated the PI3K/AKT/mTOR pathway by upregulating *PIK3AP1* transcription in CTCL. We analyzed the promoter region of PIK3AP1. The ATF/cAMP-responsive element binding protein (CREB) family, to which ATF5 belongs, share consensus binding sequences (40). Bioinformatic analysis of the PIK3AP1 promoter revealed the presence of several ATF/CREB transcription factor motifs within the region spanning -664 to -527 bp upstream of the transcription start site (**Table S3**). Chromatin immunoprecipitation (ChIP) assays confirmed the direct binding of ATF5 to this region in Myla and H9 cells (**Figure 4D**), supporting the role of ATF5 in regulating PIK3AP1 expression. As an adaptor protein, PIK3AP1 recruits the p85 subunit of PI3K and enhances p85 phosphorylation, leading to activation of PI3K/AKT/mTOR pathway activity (34–36). Immunoprecipitation confirmed interaction between PIK3AP1 and p85 in Myla and H9 cells (**Figure 4E**). In addition to the downregulation of PIK3AP1 (**Figures 4B, C**), decreased phosphorylation of p85 was observed in ATF5-KD Myla and H9 cells (**Figure 4F**), as well as decreased phosphorylation of AKT at both Thr308 and Ser473 (**Figure 4G**), confirming reduced PI3K/AKT signaling activity regulated by ATF5. Downstream of the PI3K/AKT cascade, mTORC1 promotes protein synthesis by two critical effectors, p70S6 kinase (S6K) and 4EBP1 (41, 42). Phosphorylation of both S6K at Thr389 and 4EBP1 at Thr37/46 was suppressed in ATF5-KD Myla and H9, indicating decreased mTORC1 activity (**Figure 4H**). Apart from protein synthesis, mTORC1 also facilitates a shift from OXPHOS to glycolysis, which favors macromolecular synthesis to support rapid tumor growth (43). We found that the production of lactate, an end product of glycolysis, was decreased after ATF5 knockdown (**Figure 4I**), suggesting a reduced rate of glycolysis and consistent with the decreased activity of mTORC1.

To investigate the relationship between impaired survival and decreased PI3K/AKT/mTOR activity, we treated control and ATF5-KD Myla and H9 cells with the PI3K inhibitor LY294002. PI3K inhibition remarkably impaired cell survival in both cell lines, as reported previously (44, 45). The cell viability of treated cells (OD) was normalized by the viability of the corresponding untreated cells (OD0μM). ATF5-KD Myla and H9 cells showed less pronounced decreases in cell viability (higher relative cell viability) (**Figure 4J**). This indicates that cells with ATF5-KD and decreased PI3K activation are less vulnerable to further exogeneous PI3K inhibition, compared to cells with intact PI3K activity, suggesting that ATF5 promoted CTCL survival, at least in part, by enhancing PI3K/AKT/mTOR pathway activity.

Collectively, these results showed that ATF5 transcriptionally upregulated PIK3AP1 and enhanced the PI3K/AKT/mTOR pathway to promote the survival of CTCL.

## 4 Discussion

CTCL is a malignancy arising from mature skin-homing T cells, which provides adaptive defense and participates in skin inflammation (46–48). Identifying specific molecules and mechanisms in malignant T cells enhances our understanding of their aggressive behaviors. Hyperactivation of STAT3 and STAT5 has been shown to promote the survival of malignant T cells by transcribing oncogenic cytokines and microRNAs (49–51). Advanced-stage CTCL has been associated with a skewed T helper 2 phenotype driven by GATA3 (52, 53). These findings highlight the importance of transcriptional dysregulation in CTCL, while malignant T cell-specific transcription factors in CTCL were still lacking. In our study, we identified overexpression of the transcription factor ATF5 in malignant T cells and further elucidated its role in promoting CTCL. ATF5 belongs to the ATF/cAMP response element binding (CREB) protein family, a group of basic-region leucine zipper (bZIP) transcription factors (40, 54) that play crucial roles in regulating cell differentiation and survival (55, 56). Increased ATF5 expression has been reported in aggressive chronic lymphocytic leukemia (57), follicular lymphoma (58), and childhood acute lymphoblastic leukemia (59), implying potential involvement of ATF5 in the tumorigenesis and progression of these hematopoietic malignancies. Our study not only demonstrated the specific overexpression of ATF5 on malignant T cells, but also uncovered its functional significance in CTCL. Hence, our findings established ATF5 as a tumor-specific prosurvival transcription factor, contributing to the current understanding of transcriptional dysregulation in CTCL.

ATF5 has been implicated in tumor development by transcriptional regulation of downstream genes (60–63). We found that ATF5 enhanced the survival of malignant T cells partially through the PI3K/AKT/mTOR pathway downstream of PIK3AP1. Hyperactivation of the PI3K/AKT/mTOR pathway has been reported to promote CTCL (39). Treating CTCL by targeting the PI3K/AKT/mTOR pathway has been proven effective alone (64–67) or in combination with other strategies (45, 68), whereas the treatment responses vary among individuals. Multiple factors contributed to the regulation of PI3K/AKT/mTOR pathway. Our results showed that ATF5 promotes the activation of the PI3K/AKT/mTOR axis by regulating PIK3AP1, adding to the understanding of the regulation of this pathway in CTCL. Consequently, the expression levels of ATF5 in CTCL may serve as a molecular marker indicating the effectiveness of PI3K inhibition, thereby warranting further investigation.

Cellular ER stress can be triggered by intrinsic oncogenic signals, metabolic alterations, exposure to cytotoxic drugs, and changes in the microenvironment (69, 70). Tumor cells develop tolerance to ER stress as a survival strategy against these triggering conditions (71). Although ATF5 is not an established participant in the ER stress signaling, it has been reported to affect the tolerance to ER stress through diverse mechanisms (31, 72, 73). In our study, we have observed that ATF5 protected against ER stress-induced apoptosis. However, the mechanism through which ATF5 provides this protection is yet to be fully understood, and further investigation is required to elucidate this mechanism. Several studies have highlighted the link between the PI3K/AKT/mTOR pathway and ER stress. Activation of the PI3K/AKT pathway by PIK3AP1 mediates resistance to apoptosis under ER stress (74). And drug-induced ER stress leads to apoptosis by suppressing the PI3K/AKT/mTOR pathway (75, 76). These findings support the PI3K/AKT/mTOR pathway as a pro-survival factor under ER stress, which is consistent with our results. However, other studies have proposed that aberrantly activated mTORC1 lead to the accumulation of misfolded or unfolded proteins in the ER, thereby inducing ER stress (77, 78). Therefore, the interaction between the PI3K/AKT/mTOR pathway and ER stress remains controversial in different cellular contexts (79). Crosstalk between the mTOR pathway and ER stress is involved in cancer drug resistance (80, 81). Our results suggested a positive correlation between poor treatment response, increased PI3K/AKT/mTORC1 activity and ER stress resistance mediated by ATF5. Therefore, it remains to be elucidated whether the PI3K/AKT/mTOR activity and ER stress resistance enhanced by ATF5 are related and how they might contribute to the survival of malignant T cells during anticancer treatment.

In accordance with previous studies showing that ATF5 participates in the stress response (82, 83), we have demonstrated that ER stress inducers promote ATF5 expression in CTCL cell lines, but the specific mechanisms responsible are not fully understood. One possible explanation is the involvement of the unfolded protein response (UPR), which is activated in response to ER stress to restore ER homeostasis (84). Notably, activating transcription factor 4 (ATF4), a transcription factor downstream of UPR, has been reported to directly induce ATF5 expression in response to ER stress (31, 85). However, the complexity of ER stress signaling pathways leave open the possibility of additional mechanisms contributing to ATF5 upregulation, which should be investigated in future studies.

We found than ATF5 exhibited the highest expression in malignant T cells, while it was also prominently detected in macrophages in CTCL microenvironment, whose clinical relevance remains largely unknown. There has been a report of ATF5’s upregulation and involvement in THP-1 cell differentiation *in vitro* (86). The status of tumor microenvironment is closely related to treatment response in patients (87). Further investigations into the expression of ATF5 across diverse cell types could facilitate a comprehensive understanding of ATF5’s role in CTCL and its associated microenvironment.

In conclusion, our results revealed the tumor-specific upregulation of the transcription factor ATF5, which positively correlated with worse treatment responses. Upregulated ATF5 promoted CTCL through the PI3K/AKT/mTOR pathway and conferred resistance to ER stress in CTCL. Our study uncovered the functional roles of ATF5 in promoting CTCL and provided new insights into the distinct features between malignant T cells and normal T cells.

## Data availability statement

The datasets presented in this study can be found in online repositories. The names of the repository/repositories and accession number(s) can be found below: https://doi.org/10.6084/m9.figshare.24151839.v1.

## Ethics statement

The studies involving humans were approved by Peking University First College Hospital Ethics Committee. The studies were conducted in accordance with the local legislation and institutional requirements. The participants provided their written informed consent to participate in this study. The animal study was approved by Institutional Animal Care and Use Committee of Peking University First Hospital. The study was conducted in accordance with the local legislation and institutional requirements.

## Author contributions

MC: Investigation, Validation, Writing – original draft, Visualization. PL: Methodology, Investigation, Writing – original draft. XL: Data curation, Writing – original draft, Resources. FL: Data curation, Writing – original draft, Methodology. YQ: Data curation, Writing – original draft, Formal Analysis. PT: Funding acquisition, Supervision, Writing – review & editing. YW: Supervision, Writing – review & editing, Funding acquisition, Project administration.
