# Supplementary material for: ATF5 promotes malignant T cell survival through the PI3K/AKT/mTOR pathway in cutaneous T cell lymphoma
Source: Front Immunol. 2023 Dec 22;14:1282996. doi: 10.3389/fimmu.2023.1282996 (PMC10786347; doi:10.3389/fimmu.2023.1282996)
Supplement: Supplementary file 1 [file DataSheet_1.pdf]

frontiers

Supplementary Material

ATF5 promotes malignant T cell survival through the PI3K/AKT/mTOR pathway in cutaneous T cell lymphoma.

Mengzhou Cao, Xiangjun Liu, Fengjie Liu, Yao Qin, Yang Wang*

*** Correspondence:** Yang Wang: yangwang_dr@bjmu.edu.cn

1 Supplementary Materials and Methods

1.1 Cell line RNA sequencing and gene expression analysis

For cell line RNA sequencing, total cell RNA was assessed by Bioanalyzer 2100 system for amount and integrity. After library preparation, sequencing was conducted by the Illumina NovaSeq 6000. Clean reads were aligned to the human hg38 reference genome using Hisat2. The read numbers mapped to each gene were counted using FeatureCounts. The FPKM of each gene was calculated based on the length of the gene and read count mapped to this gene. Differential expression analysis was conducted using DESeq2.

1.2 Patient outcome assessment

Disease progression was defined as progression to a more advanced TNMB classification or death owing to disease(1). TTNT was calculated as the time from the date of treatment initiation and the date of initiation of the subsequent next treatment (2).

1.3 Immunohistochemistry and immunofluorescence

Heat-induced antigen retrieval was performed on paraffin-embedded sections, and slides were incubated with antibodies against ATF5 (HPA, rabbit), or ATF5 (HPA, rabbit) with CD3 (Abcam, mouse). For immunohistochemistry, the 2-step plus® Poly-HRP Anti-Mouse/Rabbit IgG Detection System (ZSGB-BIO) was used. For immunofluorescence, Alexa Fluor 488- conjugated anti-rabbit secondary antibody, Fluor 594-conjugated anti-mouse secondary antibody (Invitrogen) and DAPI were used. Representative images were captured by NanoZoomer S60 (Hamamatsu) from regions where CD3+ cells were enriched in five patients. Three CD3+ cell-rich regions were taken from each patient, and the number of cells with nuclear ATF5 staining among CD3+ T cells was manually counted.

1.4 Western blot analysis

Cells were harvested and lysed with the Whole Cell Lysis Assay (KeyGEN) or Nuclear and Cytoplasmic Protein Extraction Kit (Beyotime). SDS–PAGE immunoblot analyses were performed with the following antibodies: anti-ATF5 (HPA), anti-β-actin (CST), anti-HDAC1 (Abcam), anti-α-tubulin (Abcam), anti-Phospho-p70 S6 Kinase (Thr389) (CST), anti-p70 S6 Kinase (CST), anti-Phospho-4E-BP1 (Thr37/46) (CST), anti-4E-BP1 (CST), anti-Phospho-PI3 Kinase p85 (Tyr458)

(CST), anti-PI3 Kinase p85 (CST) and anti-PIK3AP1 (HPA). The density was measured using ImageJ.

1.5 Real-time quantitative reverse transcription PCR (qRT–PCR)

Total RNA was extracted from cells using the RNeasy Mini kit (QIAGEN) and reverse transcribed with PrimeScript™ RT Master Mix (Perfect Real Time) (Takara). qRT–PCR was performed using PowerUp^{TM} SYBR^{TM} Green Master Mix (Thermo Fisher) with glyceraldehyde-3-phosphate dehydrogenase (GAPDH) as the internal control. The expression levels were calculated as copies of specific genes per 10000 copies of GAPDH. Relative expression levels were calculated as expression levels relative to control groups. The sequences of the primer pairs used are listed in Supplementary Table S1.

1.6 Colony-forming cell (CFC) and cell cycle assays

Colony-forming cell assays were performed in semi-solid methylcellulose cultures (StemCell) following the manufacturer's instructions. After 14 days of culture, colony numbers were counted. The colonies were categorized as follows: large colonies (those with a diameter > 60 μm), medium colonies (30 to 60 μm), and small colonies (< 30 μm)(3). Pictures were taken by Cytation 5 Cell Imaging Reader (BioTek).

For cell cycle analysis, cells were fixed with 70% ethanol, treated with DNAse-free RNAse, and stained with propidium iodide. Cell cycle distribution were measured by flowcytometry and analyzed by Modfit.

1.7 Mouse xenograft models

A total of 8 six-week-old female NOD/SCID interleukin-2 receptor γ-chain–deficient (NSG) mice were purchased from Beijing Vital River Company. Mice were kept in specific pathogen-free conditions, and 5×10^6 cells were subcutaneously injected into the right flank. The tumors were measured with calipers every two to three days, and tumor volume (TV) was calculated as $(\text{length}\times\text{width})^2/2$. The protocol was reviewed and approved by the Institutional Animal Care and Use Committee at Peking University First Hospital.

1.8 Chromatin immunoprecipitation (ChIP) and protein immunoprecipitation (IP)

ChIP assays were conducted using the iDeal ChIP-seq kit for transcription factors (Diagenode). ChIP-grade antibody against Flag (Sigma–Aldrich) or IgG (CST) was used for immunoprecipitation. The primer pair sequences used in qPCR are listed in Supplementary Table S1.

For protein IP, cells were harvested and lysed with Pierce™ IP Lysis Buffer. Antibody against p85 (CST) or IgG (CST) were used for immunoprecipitation with Pierce™ protein A/G agarose. The eluted product was detected using western blot analysis with antibody against p85 (CST) or PIK3AP1 (HPA).

2 Supplementary Figure

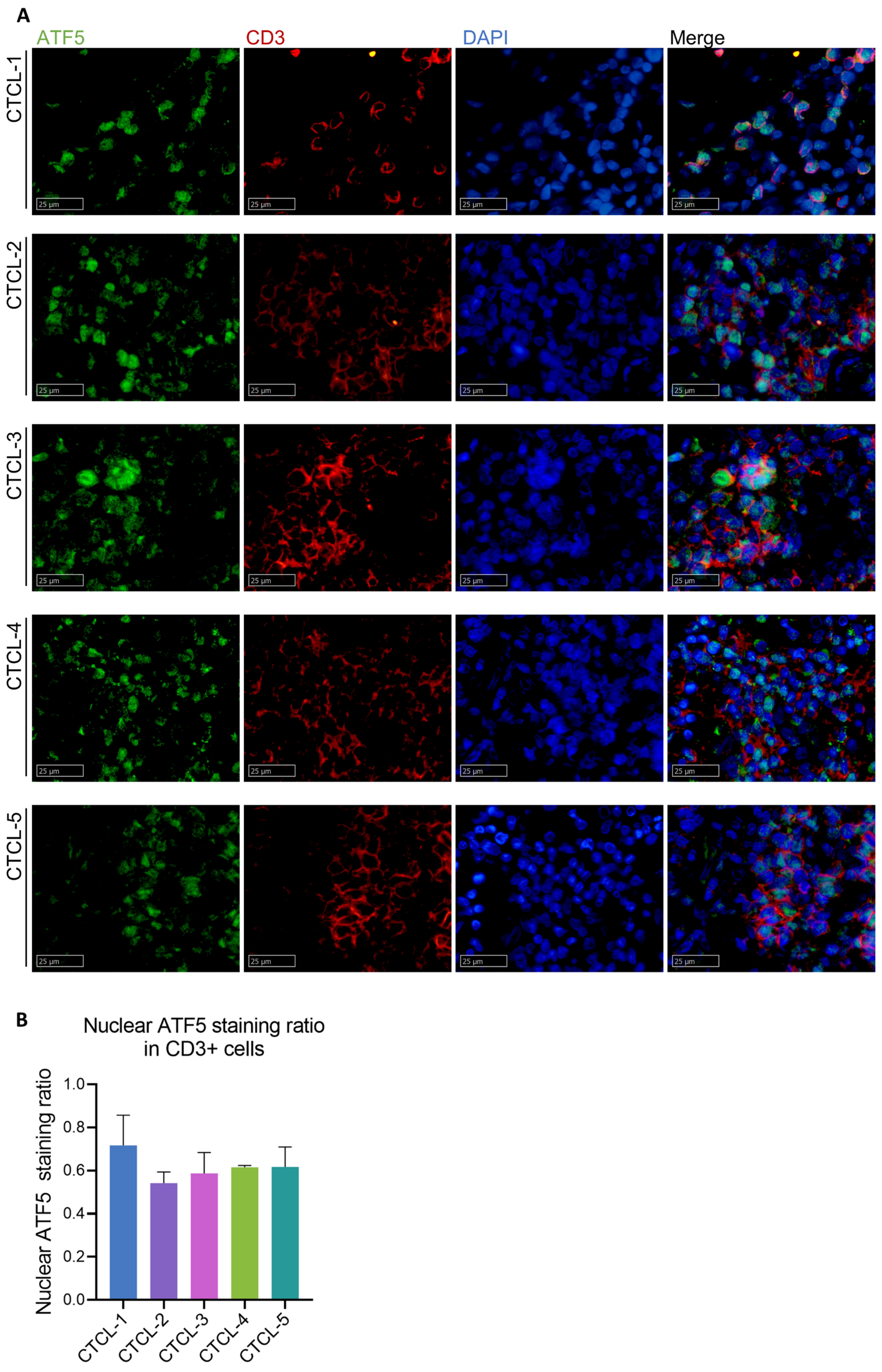
A
ATF5
CD3
DAPI
Merge
CTCL-1
CTCL-2
CTCL-3
CTCL-4
CTCL-5
25 µm
B
Nuclear ATF5 staining ratio
in CD3+ cells
Nuclear ATF5 staining ratio
1.0
0.8
0.6
0.4
0.2
0.0
CTCL-1
CTCL-2
CTCL-3
CTCL-4
CTCL-5

Supplementary Figure 1. Immunofluorescence staining of ATF5 in five CTCL patients. (A) Representative images of immunofluorescence costaining of ATF5 (green) and CD3 (red) in paraffin-embedded tissues from five CTCL patients (CTCL-1 to -5). DAPI (blue) was used to visualize cell nuclei. Original magnification ×800, scale bar=25 µm. (B) The ratio of cells with nuclear ATF5 staining in CD3+ cells.

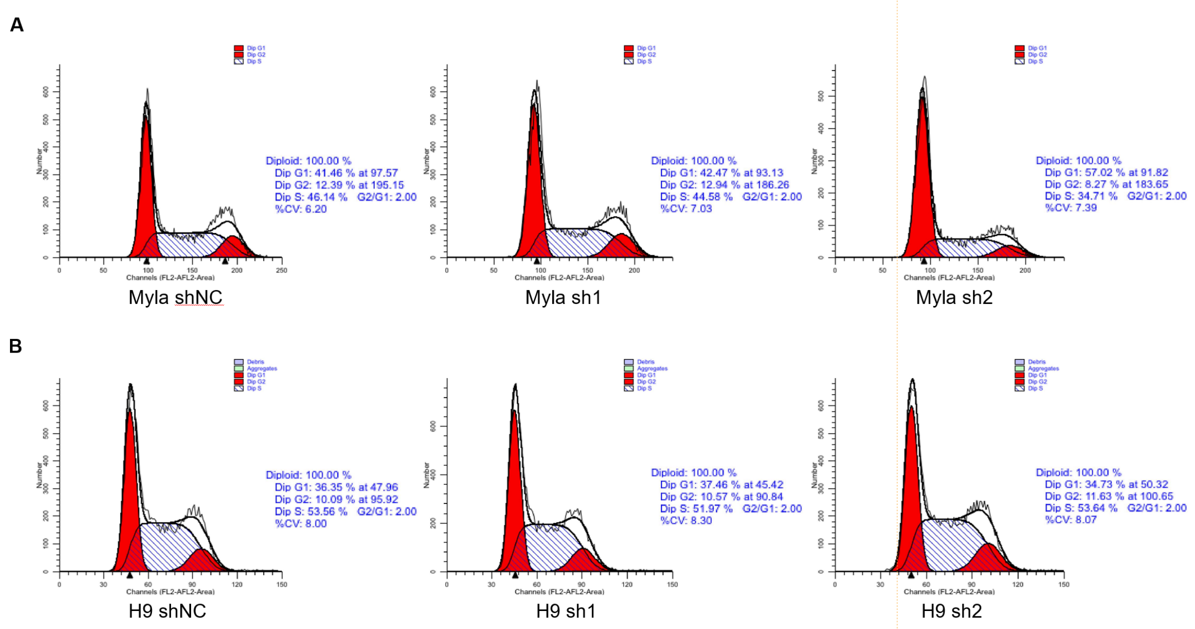

Supplementary Figure 2. ATF5 knockdown did not influence the cell cycle in CTCL. (A, B) Cell cycle analysis of control (shNC) and ATF5-KD (sh1, sh2) Myla (A) and H9 (B) cells. Proportions of cell counts in the G1, G2, and S stages are depicted and labeled aside.

3 Supplementary Tables

Supplementary table S1. Primer pair sequcences and shRNA sequences.

Supplementary Table S2. Annotated pathways from genes positively correlated with ATF5.

Supplementary table S3. Predicted binding site on PIK3AP1 promoter for ATF/CREB family transcription factors.

4 Supplementary references

1. Agar NS, Wedgeworth E, Crichton S, Mitchell TJ, Cox M, Ferreira S, et al. Survival outcomes and prognostic factors in mycosis fungoides/Sézary syndrome: validation of the revised International Society for Cutaneous Lymphomas/European Organisation for Research and Treatment of Cancer staging proposal. J Clin Oncol. 2010;28(31):4730-9.

2. Olsen EA, Whittaker S, Willemze R, Pinter-Brown L, Foss F, Geskin L, et al. Primary cutaneous lymphoma: recommendations for clinical trial design and staging update from the ISCL, USCLC, and EORTC. Blood. 2022;140(5):419-37.

3. Ringrose A, Zhou Y, Pang E, Zhou L, Lin AE, Sheng G, et al. Evidence for an oncogenic role of AHI-1 in Sezary syndrome, a leukemic variant of human cutaneous T-cell lymphomas. Leukemia. 2006;20(9):1593-601.
